# Tracheal T-Tube Stent for Laryngotracheal Stenosis: Ten Year Experience

**Published:** 2014-01

**Authors:** Arjun Dass, Nitin M Nagarkar, Surinder K Singhal, Hitesh Verma

**Affiliations:** 1 *Department of Otorhinolaryngology, Government Medical College and Hospital, Chandigarh, India.*

**Keywords:** Direct laryngoscopy, Laryngotracheal stenosis, Tracheal T-tube, Tracheostomy

## Abstract

**Introduction:**

The purpose of this retrospective study was to evaluate the outcome following stenting over a period of 10 years in patients with chronic laryngotracheal stenosis.

**Materials and Methods:**

Between 2000–2010, out of 111 patients with laryngotracheal trauma, 71 underwent tracheal T-stenting for laryngotracheal stenosis in the Department of Otorhinolaryngology at the Government Medical College and Hospital, Chandigarh, India. All 71 patients underwent stenting by tracheal T-stent through an external approach. The follow-up period ranged from 3–10 years (mean, 3.2 years). The tracheal T-stent was removed after a minimum period of 6–12 months.

**Results:**

The majority of patients in this study were aged less than 10 years or between the ages of 20–30 years. A pre-operative tracheostomy (emergency or elective) was performed in all patients. of 71 patients, decannulation was not possible in six (8%).

**Conclusion:**

Management of laryngotracheal stenosis is a challenging problem that demands a multidisciplinary approach from surgical teams well trained in this field. The ideal treatment option should be individualized according to patient characteristics. The use of silastic stents has both advantages and disadvantages.

## Introduction

Laryngotracheal stenosis is a challenging problem in the field of laryngology. Stenosis of the trachea was recorded in the literature by Colles in 1886 and laryngeal stenosis was also recognized in the 19^th^ Century by O’Dwyer, in 1887 and 1894. In the majority of patients, acquired stenosis of the larynx and trachea is due to accidental trauma, prolonged intubations, or tracheostomy (1). This can be attributed to the increased number of vehicles on the road and increasing use of mechanical ventilation in the intensive care unit, with or without tracheostomy (2). Congenital stenosis, caustic injury, and granulomatous diseases are also etiological factors in laryngotracheal stenosis (3). In the otolaryngological community, the traditional treatment for laryngotracheal stenosis has been laryngofissure and laryngotracheal reconstruction (4). Although numerous articles describe various treatment modalities, no standard approach to laryngotracheal stenosis currently exists. There are two basic modalities available; i.e., endoscopic or external (5). The endoscopic approach includes traditional dilatation, laryngeal microsurgery, laser-assisted excision, and endoscopic stent placement (6-8). External surgical reconstruction, on the other hand, is recommended when conservative efforts to establish a satisfactory airway are inappropriate or have failed (9-13). The indication for each treatment modality is not yet clearly defined. In the present retrospective study, our experience and outcomes following use of a stent in cases of chronic laryngotracheal stenosis (Myer-Cotton Grades III and IV) are discussed.

## Materials and Methods

**Fig 1:**
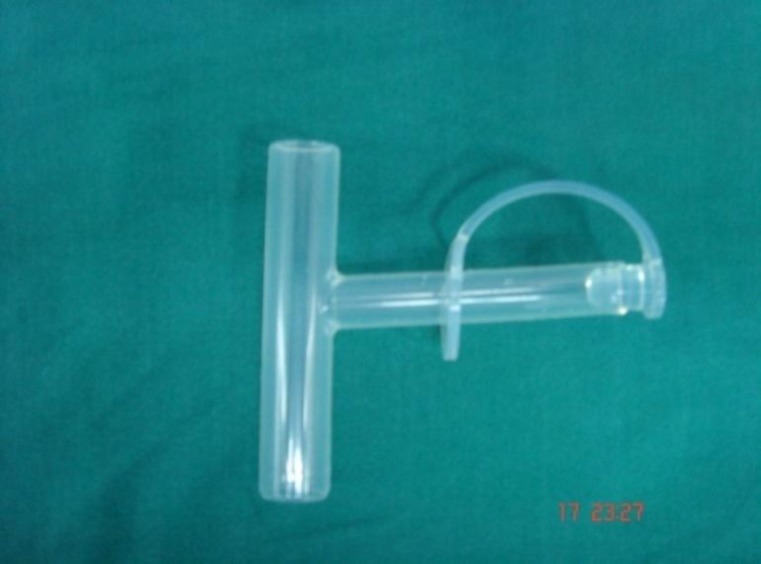
Tracheal T tube stent

One hundred and eleven patients with chronic laryngotracheal stenosis were treated at the Department of Otorhinolaryngology, Government Medical College and Hospital, Chandigarh, India from January 2000 to December 2010. A laryngotracheal stent (tracheal T-tube; Fig 1) was inserted in 71 patients, while 40 patients were treated by endoscopic methods; i.e., CO_2 _laser application, dilatation, and ledge removal. A patient with glottic, glottis–subglottic stenosis was excluded from this study. This paper focuses on the 71 patients treated with laryngotracheal stenosis. The data were retrospectively collected from the Medical Record Department. The follow-up period ranged 3–10 years (mean follow-up, 3.2 years). In this study, there were 48 males and 23 females. The age range was from 2–80 years (median age, 33.35 years) (Table. 1). 

**Table 1:** Age and Sex Distribution

**Age**	**Male**	**Female**
Less than 10 years	10	5
11-20 years	9	4
21-30 years	13	11
31-40 years	8	2
41-50 years	1	
51-60 years	3	1
61-70	3	
71-80	1	

**Fig 2:**
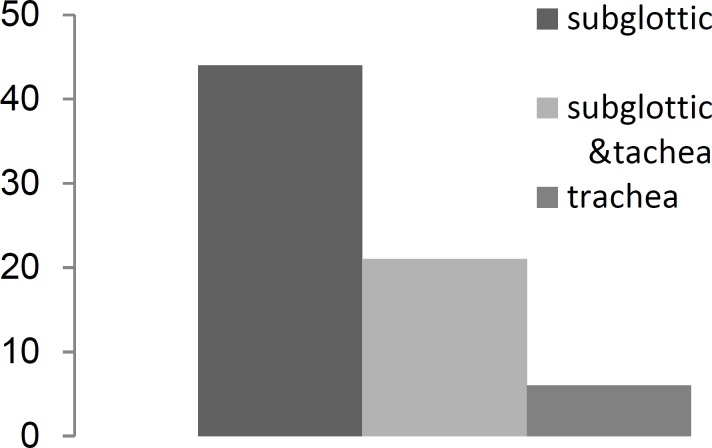
Area of Stenosis

All 71 patients were tracheostomized. The stenosis was grade III or grade IV in all cases (Myer-Cotton grading). The stenosis was subglottic in 44 patients (>5 mm below vocal cords), subglottic and tracheal in 21 patients, and tracheal in six patients (Fig 2). 

The etiology of laryngo-trachea stenosis was accidental trauma in the form of roadside accident or strangulation and intubation trauma in the majority of the cases.

***Patient Evaluation***

All patients of any age group and either sex with a history of respiratory difficulty due to trauma or prolonged intubation, or tracheostomized outside for the same, were admitted. Tracheostomy was performed on the patient as and when required. Routine blood investigations (i.e. hemogram, coagulogram, serum electrolytes, and renal function tests) were performed in all patients. Radiological investigations included X-ray soft tissue neck lateral view and X-ray chest PA view. Direct laryngoscopic examination was performed under general anesthesia. Patients with grade I or II stenosis were subjected to endoscopic procedures, whereas patients with grade III or IV stenosis were further evaluated to ascertain the length of the stenotic segment using an endoscopic method. None of patients failed under the endoscopic method. Patients having complete stenosis were advised to have a computed tomography (CT) scan to ascertain the length of the stenotic segment. Three patients presented with a magnetic resonance image (MRI) of the neck. 

***Operative Technique***

All patients underwent direct laryngoscopy and tracheoscopy under general anesthesia. The larynx was also examined under the microscope. Saline/adrenaline (1:100,000) was infiltrated subcutaneously into the skin. A transverse cervical skin incision was marked using a marker pen, and the skin flaps were raised. 

The trachea and larynx were exposed. An oblique incision was made on the anterior wall of the trachea to expose the stenotic segment. When required, the incision was extended on to the cricoid cartilage. The major part of fibrous tissue of the stenotic segment was removed by cautery, whereas fine work was carried out by CO2 laser as applied using a hand piece. The stent (tracheal T-stent) was fixed across the stenotic segment. 3-0 vicryl sutures were placed over the cut end of the stenotic segment covering the stent to stabilize the tube. Direct laryngoscopy was performed to confirm the position of the stent. The strap muscles were sutured over the segment, and the wound was then sutured in layers. A course of broad-spectrum antibiotics was given for a period of 7 days. Regular suction was undertaken through the tracheal T-stent. 

**Fig 3:**
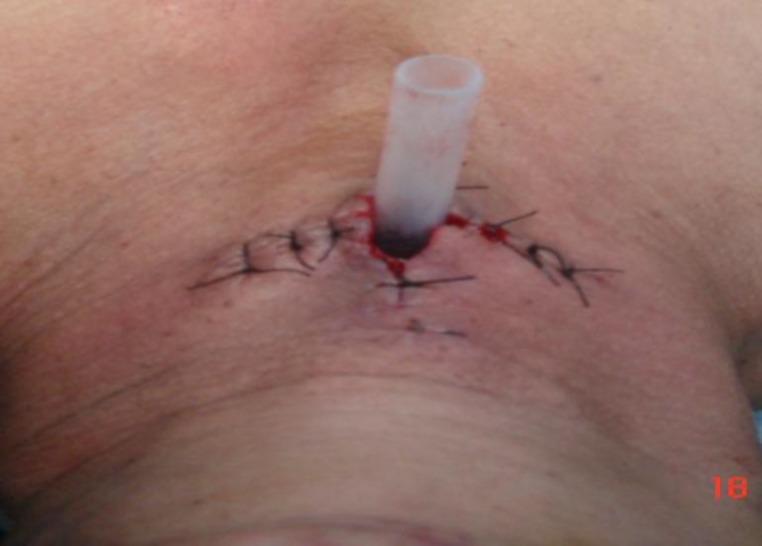
Post surgical view (inferiorly replaced)

The skin sutures were removed on the seventh post-operative day, while a follow-up endoscopy was performed after a period of 1 month. If the T-stent was found to be touching the cords, it was displaced inferiorly after removing the lower part of stoma, and tube was stabilized (Fig. 3). 

**Fig 4:**
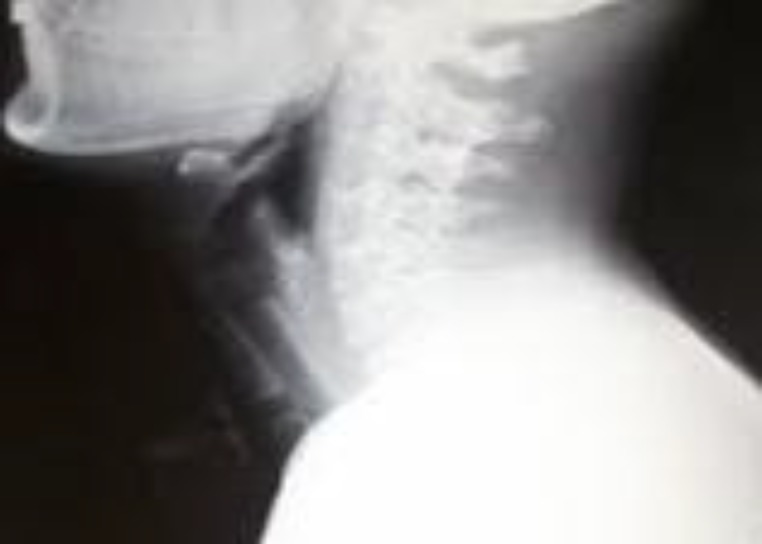
**-**X Ray S/T/N Lateral View (Tracheal T Tube in situ

The tracheal T-stent was removed after a minimum period of 6–12 months following complete evaluation (Fig. 4). 

The T-stent was removed earlier in children (mean, 3.6 months) compared with adults (mean, 5.8 months). Removal of stent was also early in cases of penetrating trauma as compared with blunt trauma. Examination of the larynx, stenotic segment, and stoma was carried out under general anesthesia. Depending on the age of the patient, a small tracheostomy tube was inserted for a period of 2–3 weeks and then decannulation was performed. Scar revision was carried out as requested.

## Results

**Fig 5:**
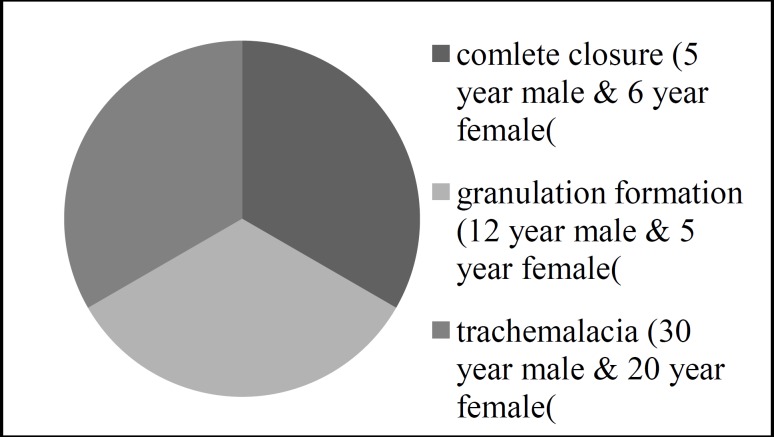
Causes, age & sex distribution in failure cases

In this series, out of 111 patients who attended the ear, nose and throat outpatient department (ENT OPD) with chronic laryngotracheal stenosis, 71 patients underwent surgery with opening of the stenotic segment and stenting with tracheal T-stent. The length of the stenotic segment varied from 5–40 mm. Fifteen patients were under the age of 10 while 24 were aged between 21–30 years (Table. 1). In the age group below the 10 years, the most common indication was post-intubation subglottic stenosis, similar to a previous study reported by Triglia et al (14). In the 21–30 year age group, external trauma was found to be the commonest etiology, while prolonged intubation was the most common cause, as previously found in a study by Mohammed et al (2). A pre-operative tracheostomy (emergency or elective) was performed in all patients. There was no perioperative or post-operative mortality; however, five patients developed mild surgical emphysema and were treated conservatively. Out of 71 patients, we were not able to decannulate six (8%), compared with a success rate of 100% (4,15), 93% (16), and 95% (17) in previously reported studies. The cause was tracheomalacia in two patients, granulation formation between stent and glottis in two patients, and complete closure of the trachea in two patients (Fig. 5). 

In patients with tracheomalacia, reconstru-ction of the trachea was carried out with a rib graft along with tracheal T-tube stenting. In patients with granulation formation, the laryngeal stent was reinserted at a lower position with application of a laser to remove granulation. In one patient, complete closure resection of the stenotic segment and end-to-end anastomosis was planned (18,19); whereas, one further patient was lost to follow-up. 

## Discussion

Management of laryngotracheal stenosis is a challenging problem that demands a multidisciplinary approach from surgical teams well trained in this field. The aim of any treatment modality is, in order of priority: airway patency (1,2), glottic competence for airway protection against aspiration, and acceptable voice quality (3,20). Permanent tracheotomy, the oldest and simplest treatment method, has limitations, including an inability to vocalize without occluding the stoma, inherent disfigurement associated with wearing of the tracheotomy tube, and the inability to engage in certain recreational activities (e.g., swimming). Decannulation and closure of a preliminary tracheotomy is thus yet another goal of modern therapy addressing airway stenosis (21). Various forms of treatment described for laryngotracheal stenosis include laser, repeated endoscopic dilatations, cryosurgery, prolonged stenting, laryngotracheal reconstru- ction, and segmental resection with end-to-end anastomosis. The ideal treatment option should be individualized based on patient characteristics, as each procedure has its own advantages and disadvantages (22). Definitive laryngotracheal surgery should only be attempted after edema and inflammation have subsided for better anastomotic results. The use of silastic stents has both advantages and disadvantages. The ‘tracheal stent’ provides an excellent airway (23-25). The upper stem of the tracheal T-stent provides appropriate support to the subglottis and permits immediate post-operative phonation. Stents have been proposed to protect laryngeal patency from the contracture of scar tissue, to promote the development of a new epithelial cover, and to prevent mechanical disruption caused by the movements of swallowing and breathing during healing (26,27). Stenting remains a relatively conservative treatment, is successful in a proportion of cases, and does not preclude the possibility of future reconstructive surgery if it fails. The tracheal T-stent initiates little or no tissue reaction unless it touches the undersurface of the vocal cord; as occurred in two cases in this study due to the close proximity of the stenotic segment with the vocal cord. This serves as both a stent and a tracheostomy tube. The intraluminal portion is of sufficient density and thickness to support a reconstituted stenotic larynx and trachea. Mucus and crusts do not readily adhere to the smooth surface of the silicone material. Most of the time, the soft T-stent remains plugged, thus allowing respiration and phonation while maintaining the airway (28). However, recognized complications include problems with stent fixation leading to subsequent migration, and luminal blockage caused by impacted secretions, or the overgrowth of inflammatory granulation tissues (29). 

The challenge of providing trouble-free stenting demands much patience, a degree of lateral thinking, and the occasional inspiration. We were not able to decannulate all patients, even after different surgical treatment modalities were applied in our patients. This may be due to variability in the healing process in different patients, closeness of the stenotic segment to the vocal cord, variability in the expertise of the surgeon, and the need more strength and less tissue reacting stenting material, even after the touching of a vocal cord. 

## Conclusion

The management of laryngotracheal stenosis is a challenging problem that demands a multidisciplinary approach performed by surgical teams well trained in this field. The ideal treatment option should be individualized based on patient characteristics. Stenting remains a relatively conservative treatment, is successful in a proportion of cases, and does not preclude the possibility of future reconstructive surgery if it fails.
